# Supplementary material for: Lignin induced iron reduction by novel sp., Tolumonas lignolytic BRL6-1
Source: PLoS One. 2020 Sep 17;15(9):e0233823. doi: 10.1371/journal.pone.0233823 (PMC7497984; doi:10.1371/journal.pone.0233823)
Supplement: S1 Raw images — Silver stained SDS-PAGE of T. lignolytica BRL6-1 secretome cultured in unamended conditions (lanes 2–4) and lignin-amended (lanes 7–9). Lanes 1 and 10 are Precision Plus Protein Dual Color Standards (250-10kDa). Lanes 4 and 5 are abiotic controls of lignin-amended conditions. Image was taken with flat bed scanner. Figure in manuscript was submitted as gray scale with ladder lanes cropped (X) and substituted for labels instead. Figure is labeled as Fig 7. (PDF) [file pone.0233823.s001.pdf]

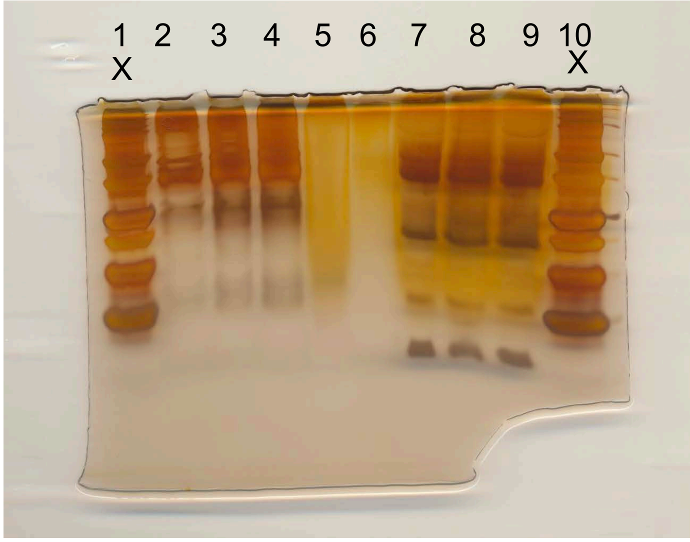

S1 Raw Images: Undoctored image of silver stained gel. Silver stained SDS-PAGE of *T. lignolytica* BRL6-1 secretome cultured in unamended conditions (lanes 2-4) and lignin-amended (lanes 7-9) . Lanes 1 and 10 are Precision Plus Protein Dual Color Standards (250-10kDa). Lanes 4 and 5 are abiotic controls of lignin amended conditions. Image was taken with flat bed scanner. Figure in manuscript was submitted as gray scale with ladder lanes cropped (X) and substituted for labels instead. Figure is labeled as Fig 7.
